# A coarse‐refine segmentation network for COVID‐19 CT images

**DOI:** 10.1049/ipr2.12278

**Published:** 2021-11-18

**Authors:** Ziwang Huang, Liang Li, Xiang Zhang, Ying Song, Jianwen Chen, Huiying Zhao, Yutian Chong, Hejun Wu, Yuedong Yang, Jun Shen, Yunfei Zha

**Affiliations:** ^1^ School of Data and Computer Science Sun Yat‐Sen University Guangzhou China; ^2^ Department of Radiology Renmin Hospital of Wuhan University Wuhan China; ^3^ Department of Radiology Sun Yat‐Sen Memorial Hospital Sun Yat‐Sen University Guangzhou China; ^4^ School of Systems Sciences and Engineering Sun Yat‐Sen University Guangzhou China; ^5^ Department of Radiology The Third Affiliated Hospital of Sun Yat‐Sen University Guangzhou China

## Abstract

The rapid spread of the novel coronavirus disease 2019 (COVID‐19) causes a significant impact on public health. It is critical to diagnose COVID‐19 patients so that they can receive reasonable treatments quickly. The doctors can obtain a precise estimate of the infection's progression and decide more effective treatment options by segmenting the CT images of COVID‐19 patients. However, it is challenging to segment infected regions in CT slices because the infected regions are multi‐scale, and the boundary is not clear due to the low contrast between the infected area and the normal area. In this paper, a coarse‐refine segmentation network is proposed to address these challenges. The coarse‐refine architecture and hybrid loss is used to guide the model to predict the delicate structures with clear boundaries to address the problem of unclear boundaries. The atrous spatial pyramid pooling module in the network is added to improve the performance in detecting infected regions with different scales. Experimental results show that the model in the segmentation of COVID‐19 CT images outperforms other familiar medical segmentation models, enabling the doctor to get a more accurate estimate on the progression of the infection and thus can provide more reasonable treatment options.

## 1 INTRODUCTION

The novel coronavirus disease 2019 (COVID‐19) is spreading quickly in the world. More than ten million people were infected, among whom hundreds of thousands died since 2019 [1]. COVID‐19 is caused by a kind of severe acute respiratory syndrome coronavirus, which can spread fast to other persons and dramatically increasing the number of infected people. Thus, it's critical to find out a quick way to make diagnoses and give appropriate treatments. Unfortunately, there are no effective drugs or vaccines for COVID‐19 patients, so doctors must implement different treatment plans according to the conditions of patients. However, it is challenging to determine the conditions of patients due to a lack of quantified criteria.

Chest computed tomography (CT) is a convenient diagnostic tool for pneumonia, and it is widely used for detecting typical imaging features of COVID‐19 [2, 3, 4, 5]. Recently, many methods are proposed to discriminate patients with COVID‐19 from healthy or other types of patients through the features in CT [6, 7, 8, 9, 10, 11, 12, 13]. For example, a COVID‐Net [14] is proposed to detect patient with COVID‐19 from CT through a deep learning method. Kamini et al. [5] use a stack of pre‐trained deep models for ensemble learning to perform sensitive identification of COVID‐19 patients. Several classification methods [9, 15, 16] are proposed for distinguishing whether a person is infected with COVID‐19 by using CT. However, most of the above studies focused on classification, which cannot make full use of the information of chest CT and cannot provide more reasonable treatment options.

A recent review [17] concluded that chest CT imaging is sensitive to the detection of COVID‐19. The typical imaging features of patients are ground‐glass opacity, consolidation, and fibrosis [18, 19]. However, it is expensive to label these areas manually. A recent research [20] shows that the severity of progress of patients with COVID‐19 can be inferred from the area of imaging features. Therefore, accurate segmentation of medical images provides a viable way to estimate the progression of infections for designing reasonable treatments. Therefore, an effective segmentation method of lesion area is urgently needed.

The development of deep learning techniques provides strong supports for medical image segmentation. Recently, the residual attention U‐Net [21] is proposed for the automated segmentation of multiple COVID‐19 infection regions. It exploits both the residual network and attention mechanism to improve the efficacy of the U‐Net. Inf‐Net [20] can automatically recognise infected regions from CT. It improves the quality of boundary segmentation, but it is not effective in detecting small lesion areas. Therefore, those models cannot work very well in the segment of COVID‐19 lesions. The main challenges are as follows: First, some boundaries of infected regions are not evident [20, 22] and some areas of infection have low contrast with natural areas [21]. For example, ground‐glass opacity is often with low contrast in normal regions. Besides, the boundary of ground‐glass opacity is blurry and hard to recognise. Second, the shape and size of infected areas are multi‐scale, which are challenging to segment [21, 23]. Specifically, consolidations are too small, whereas fibrosis is always big. The multi‐scale infected regions increase the difficulty of segmentation significantly.

To address the above problems, in this paper, we proposed a coarse‐refine network for capturing both coarse and fine contexts, which can achieve accurate object segmentation with high‐quality boundaries. Specifically, we use the coarse‐refine architecture and hybrid loss to guide the model to predict the delicate structures with clear boundaries to address the problem of unclear boundaries. We add the atrous spatial pyramid pooling module (ASPP) [24] in the network to improve the performance in detecting infected regions with different scales.

The contributions of this paper are as follows:

1) We propose a network for the segmentation of COVID‐19 Chest CT slices. In this network, we use a coarse segmentation module to obtain a rough segment result from input images. We employ a refined module to learn the residuals between the rough segment result and the ground truth to adjust the erroneous segmentation output, especially the edge. Moreover, we add an atrous spatial pyramid pooling module to both the coarse segmentation module and the refine module so that the network can accurately detect infected regions with different scales. We adopt the hybrid loss [25] as training loss, which helps guide the model to predict the delicate structures with clear boundaries accurately.
2) We regard the encoder of the coarse segmentation module as a classifier. We put all the CT slides of a person into the classifier to determine whether the person is infected with COVID‐19. We only segment the images that are judged with COVID‐19 lesions, so the classifier can considerably speed up the network.
3) We analyse the statistics of the segmentation results and show that the segmentation results provide strong evidence for doctors to make a much more accurate estimate of the infection severity. Subsequently, the patients with COVID‐19 can receive more reasonable treatment options.

## 2 MATERIAL AND METHOD

This section starts with the dataset introduction. We describe the dataset in Section 2.1 followed by the strategy for dataset split in Section 2.2 and data augmentation section in Section 2.3. Then we introduce the architecture overview of our network in Section 2.4. The coarse segmentation module is described in Section 2.5, and the segmentation refinement module is introduced in Section 2.6. Finally, the loss function is presented in Section 2.7, and the part of the classifier is mentioned in Section 2.8.

### 2.1 Dataset

We collected the CT images of patients with COVID‐19 manually labelled from Renmin Hospital of Wuhan University and Sun Yat‐Sen Memorial Hospital. The manual labelling of the lesion area dataset includes 1060 CT slices from 226 patients with COVID‐19. The images were annotated by five experienced physicians and checked by two senior doctors, leading to 1060 images from 226 patients with COVID‐19. These data are used for the segmentation task. The annotations include three types of lesions: fibrosis, consolidation, and ground‐glass opacity (GGO). The severity of the illnesses is independently diagnosed to mild, moderate, and severe according to CT images and clinical symptoms. Table  1 shows the number of patients, CT slices, and the number of annotated numbers of lesions for three different severity levels of patients with COVID‐19.

**TABLE 1:** The number of patients, CT slices, and the number of annotated numbers of lesions for three different severity levels of patients with COVID‐19

Severity	Mild	Moderate	Severe	Total
Persons	51	75	100	226
CT slices	152	374	534	1060
Fibrosis	/	/	/	700
Consolidation	/	/	/	895
GGO	/	/	/	3103

To test the performance of the classifier, We collected another classification dataset. It is shown in Table  2. It contains 539 COVID‐19 patients' images without annotated infection regions, 78 healthy persons' CT slices, 100 patients' data with bacterial pneumonia and 219 patients' data with typical viral pneumonia. We regard the 78 healthy persons' CT slices, 100 patients' data with bacterial pneumonia and 219 patients' data with typical viral pneumonia as a class in the classification task to distinguish whether the patient is infected with COVID‐19. Those data are provided by Renmin Hospital of Wuhan University, Sun Yat‐Sen Memorial Hospital. The dataset isn't previously published, and is made available by request.

**TABLE 2:** The number of persons and CT slices of the different kinds of lung classification dataset

Type	COVID‐19	Healthy people	Bacterial pneumonia	Typical viral pneumonia
Persons	539	78	100	219
CT slices	3391	582	504	2159

### 2.2 Strategy for dataset split

Human‐level results are more meaningful than image‐level in conducting diagnosis and therapy. Hence, we split the dataset at the human level. We use the data of 226 patients with COVID‐19 that labelled lesion areas as the positive samples of the segmentation model. We regard the 78 healthy persons' CT slices, 100 patients' data with bacterial pneumonia and 219 patients' data with typical viral pneumonia as the segmentation model's negative samples because their images do not contain the infected region of COVID‐19. We get the data for the segmentation model by combining the positive and negative samples. We divide the segmentation dataset by splits of 70%/10%/20% as training, validation, and test data at the human level and carry out five‐fold cross‐validation. The number of COVID‐19 patients, the number of CT slices, and the number of various types of lesions in a fold of training set, validation set, and test set is shown in Table  3.

**TABLE 3:** The number of COVID‐19 patients, the number of CT slices, and the number of various types of lesions in a fold of training set, validation set, and test set

Datatype	Training	Validation	Test	Total
Persons	158	22	46	226
CT slices	704	127	229	1060
Fibrosis	430	82	188	700
Consolidation	622	88	185	895
GGO	2087	311	705	3103

We regard patients with COVID‐19 as a class. The CT images of healthy people and bacterial pneumonia and typical viral pneumonia as one category. Those data are used to recognise whether a person is infected with COVID‐19 from their CT slices. We divide the classification dataset by splits of 70%/10%/20% as training, validate and test data at the human level and also carry out five‐fold cross‐validation.

### 2.3 Data augmentation

As our deep learning methods are affected by the amount of data, we do data augmentation for our training data. In this data augmentation, all images are rotated by 90, 180, and 270 degrees to obtain three times more images. We also flipped the original images horizontally to produce another copy of the images. As a result, the data augmentation expands the number of images to five times, so we get 15,060 training images in every fold.

### 2.4 Overview of our network

**FIGURE 1:**
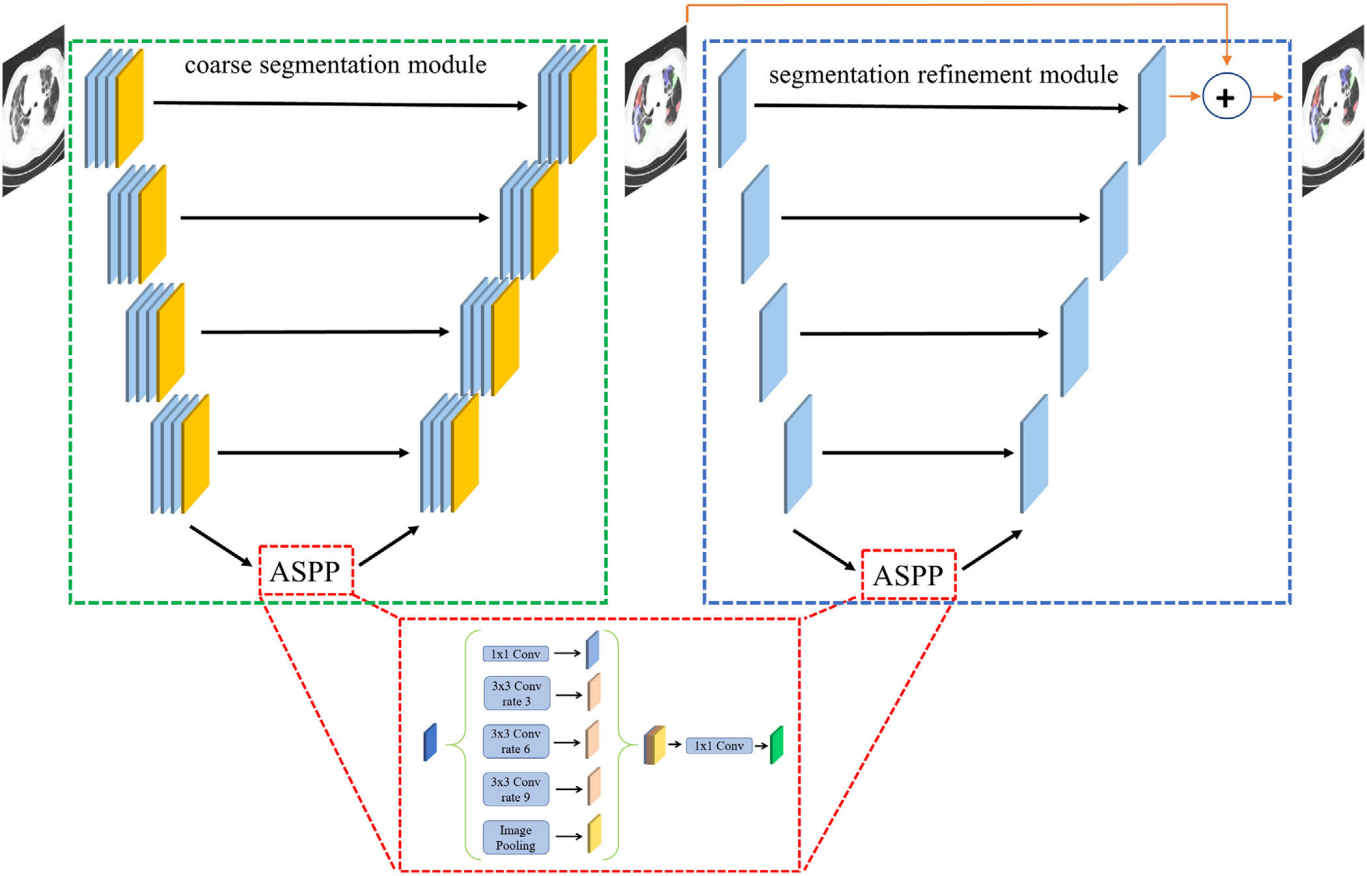
The proposed segmentation network. the green, blue, and red dotted boxes are the coarse segmentation, the segmentation refinement, and the atrous spatial pyramid pooling (ASPP) modules, respectively

In the design of our network, we consider the fact that doctors commonly locate infected regions roughly and then adjust contours to get fine annotations. Rough segmentation and the refine operation are all very important. Inspired by this, we design the network as a coarse segmentation module and a segmentation refinement module. As shown in Figure  1, the network includes two modules: the coarse segmentation module and segmentation refinement modules. They are all the U‐Net‐like architecture. The input image is firstly inputted into the coarse segmentation module to get the coarse segmentation result. Then, the coarse segmentation result is the input of the segmentation refinement modules to get the residuals between the ground truth and coarse segmentation result. The final output is obtained by adding the residuals and the coarse segmentation results.

The coarse segmentation module is used to obtain rough segmentations from input images through an encoder–decoder network based on U‐Net [26]. In this network, the rough segmentations are refined by learning the residuals between the ground truth and the result of coarse segmentation through the refinement module. We use the atrous spatial pyramid pooling (ASPP) module [24] to improve the network performance in detecting infected regions with different scales.

### 2.5 Coarse segmentation module

The coarse segmentation module is designed as an encoder–decoder network based on the U‐Net [26]. In this network, the encoder is used to extract low‐level details and high‐level global context features from original medical images and then employed to construct the segmentation results through a decoder. The architecture can simultaneously capture low‐level details and high‐level global contexts. The performs of the architecture is proved in many biomedical image segmentation tasks [26]. In our study, the encoder has an input layer and four stages comprised of se‐res‐blocks, as also used in the SE‐ResNet‐34 [27, 28]. The se‐res‐blocks make the network able to obtain higher resolution feature maps in a previous layer and thus get a better ability to extract features than general CNN blocks.

It is challenging to make accurate segmentation [21, 23]. In fact, the infected regions often come with vague boundaries [20] and have low contrasts with the background [21]. Moreover, the various shapes and sizes enhance the difficulty in segmentation. We connected the encoder and decoder by the atrous spatial pyramid pooling (ASPP) [24] to extract global information and detect objects with different scales. ASPP works well in extracting global information and detecting objects with different scales [24].

**FIGURE 2:**
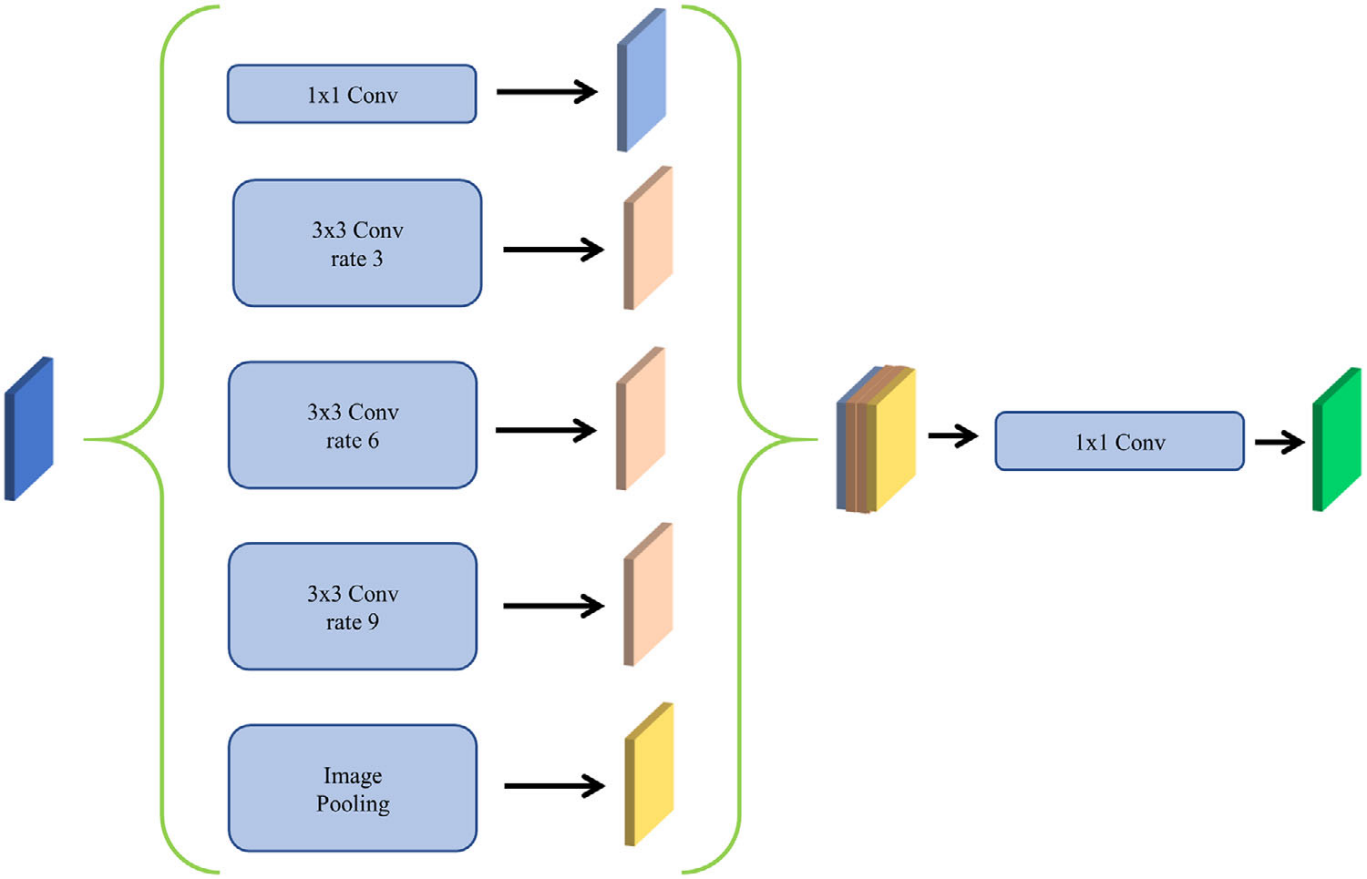
The atrous spatial pyramid pooling structure of our model

ASPP is robust to fuse semantic information of different levels [24]. As the feature maps were processed through dilated convolutions with varying expansion rates, the dilated convolutions can generate feature maps with different receptive fields. ASPP can perfectly fuse different context information produced by various receptive fields. As shown in Figure  2, ASPP comprises a 1×1 convolution, an image pooling, and three atrous convolutions with different rates in parallel. All convolution layers (the 1×1 and three atrous convolution layers) contain 64 convolution filters. The different rates (3, 6, and 9) of three atrous convolutions are used for combining high‐level semantics with low‐level details.

The structure of our decoder is symmetrical to the encoder. Each stage comprises three convolution layers and is followed by a batch normalisation and a ReLU activation function, finally followed by a bilinear up‐sampling [26] and a sigmoid function. The outputs of each stage and the corresponding stage in the encoder are concatenated to build the input feature map for the next stage.

### 2.6 Segmentation refinement module

In our network, a segmentation refinement module is designed to refine the coarse predictions by learning its difference from the ground truth, similar to previous studies [25, 29].

It has been proved that the residual refinement module has good detection ability on the edge part [25, 29]. Residual refinement module (RRMLC) [29] is proposed for boundary refinement by employing the local context, but its receptive field is too small. It [30] uses different kernel sizes and dense connections to capture multi‐scale information. RRM [25] adopts a deeper network to capture high‐level context. However, it is difficult for those modules to get high‐level information and low‐level details to refine the coarse segmentation result.

**FIGURE 3:**
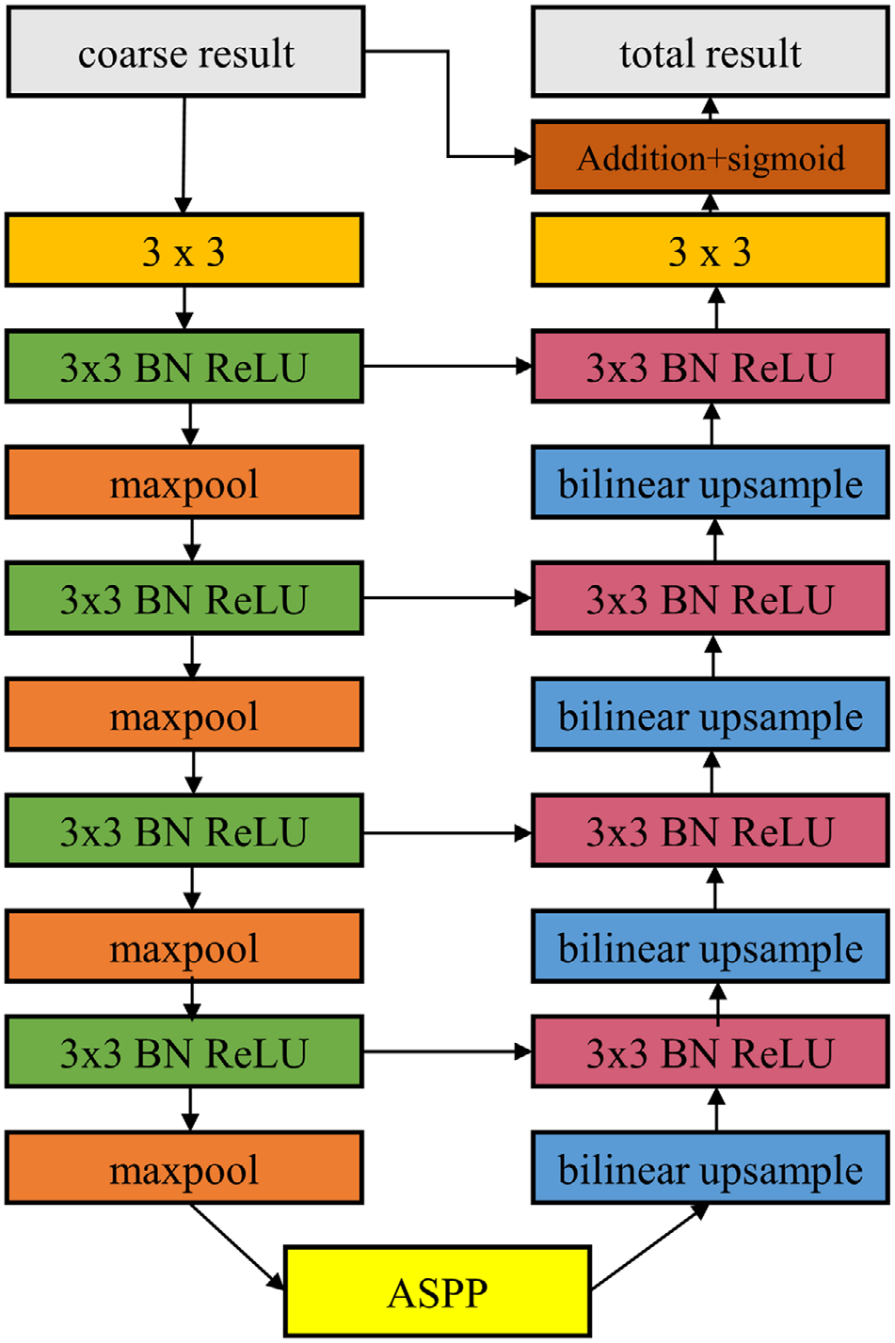
The structure of the segmentation refinement module

To improve the accurate boundary and region in coarse segmentation, we developed a segmentation refinement module based on RRM [25]. As shown in Figure  3, we proposed a refine module based on (RRM). The architecture of the segmentation refinement module is similar to our coarse segmentation module, but it is simpler. It consists of an encoder, an ASPP module, and a decoder. Same as the coarse segmentation module, both encoder and decoder have four stages. Every stage uses only one convolution layer and is followed by batch normalisation and a ReLU activation function. The convolution layer has 64 filters of size 3×3. The ASPP bridge is the same as the coarse segmentation module. Each stage of encoder and decoder is followed by a max‐pool layer and a bilinear up‐sample layer, respectively. Add the rough segmentation result and the refined result and then input the sum into a sigmoid function as the result of our model.

### 2.7 Hybrid loss

The hybrid loss was proposed in BASNet [25], which obtains high‐quality regional segmentation and clear boundaries. It combines binary cross‐entropy (BCE) [31], structural similarity (SSIM) [32], and IoU losses [33]. It expects to learn the pixel‐level, patch‐level and map‐level information from the ground truth. We use hybrid loss rather than explicit boundary losses [32, 34] mainly because injecting the goal of precise boundary prediction in the hybrid loss may help to reduce sham error from cross propagating the information learned on the boundary.

The hybrid loss [25] is defined as:

(1) $$l=l_{\text{bce}}+l_{\text{ssim}}+l_{\text{iou}}$$

Where $l_{\text{bce}},l_{\text{ssim}}$ and $l_{\text{iou}}$ denote BCE loss [31], SSIM loss [32] and IoU loss [33], respectively.

BCE loss is pixel‐level because it does not consider the local neighbourhood's labels and the weights of all pixels are equal. It is helpful for the convergence of all pixels. SSIM loss is patch‐level because it considers an adjacent neighbourhood for each pixel. It assigns a higher weight to the edge so that it helps to optimise the boundary segmentation. IoU loss is a map‐level measure because as the foreground's forecast confidence grows higher, the foreground's loss will gradually approach zero. We use BCE loss to maintain a smooth gradient for all pixels by combing those losses, and we utilise IoU loss to focus on the foreground. We use SSIM loss to provide further weight for the boundary to obtain a more accurate boundary prediction. In our model, we use hybrid loss at intermediate coarse segment output and the final output and add them up, then use backpropagation to optimise the parameters.

### 2.8 Classification

To enable the model to distinguish the person with COVID‐19 from a person without COVID‐19. For the propose to verify that the segmentation labelled data can improve the ability of network feature extraction in COVID‐19. We regard the encoder part of the coarse segmentation module as a classifier. The encoder part of our coarse segmentation module has an input layer and four stages comprised of basic se‐res‐blocks, and the input layer and four stages are adopted from SE‐ResNet‐34 [27, 28]. When the remaining parts of SE‐ResNet34 are also plugged into the four stages of the encoder to form a complete SE‐ResNet34 network which regard as a classifier, and the last layer is changed from a thousand categories to two categories. SE‐ResNet [27, 28] network is a very effective classification network that has strong feature extraction ability. We use it as a basic classifier here. For an input image, the classifier can directly determine whether the image contains the infected area of COVID‐19. For a series of slices of a patient, if all the slices don't contain the infected regions of COVID‐19, the model will judge that the patient is not infected with COVID‐19. Otherwise, the patient will be judged to be infected with COVID‐19. A classifier added not only classifies the patients with COVID‐19 but also significantly speeds up the operation of the network because we only input the picture into the later system for segmentation when the image contains the infected regions.

## 3 RESULT

### 3.1 Experiment setup

We ran the experiment on Ubuntu 16 with an NVIDIA TITAN RTX 1080 GPU with 11 GB of memory. We use Python 3.6 and PyTorch 1.0 to implement those algorithms. The size of all CT slices is 512×512. We use Adam optimiser to optimise all methods, and the learning rate is set to 3e‐4 by default. We train all models with a mini‐batch size of 8. We run up to 200 epochs for each method. The training monitors the loss on the validation dataset, and it will early stop if the loss increases too much. All those methods had the same training strategy and made the same data augmentation mention before.

Firstly, we train the whole segmentation network, and when the segmentation network converges, we freeze all parameters of the segmentation model. Later, we train the classification part of the coarse segmentation module except for the input layer and the first four stages until the classification model convergence.

In the test process, for each image of a patient, we first input the image into the encoder of the segmentation network to determine whether it has lesions. If it has some lesion of COVID‐19, we continue to segment it. When all the images of a patient are tested, if no CT image contained the lesion area, the model will predict that this person is not infected with COVID‐19. Otherwise, it will indicate the person is infected with COVID‐19.

### 3.2 Metrics

For the segmentation task, we use Dice coefficient [26], IOU [26], pixel precision [35], pixel recall [35] to evaluate the CT segmentation task. Those metrics are always used in the medical segmentation task. They can be calculated as follows:

(2) $$\text{Dice}=\frac{2\left|G\cap P\right|}{\left|G\right|+\left|P\right|}$$

(3) $$\text{IOU}=\frac{\left|G\cap P\right|}{\left|G⋃P\right|}$$

(4) $$\text{Pixel}\;\text{Precision}=\frac{\left|G\cap P\right|}{\left|P\right|}$$

(5) $$\text{Pixel}\;\text{Recall}=\frac{\left|G\cap P\right|}{\left|G\right|}$$

where G is the set of ground truth pixels of the lesions and P is the set of predicted pixels. |G∩P| is the number of elements of the intersection between ground truth pixels and predicted pixels, and |G| and |P| are the number of elements of the set of ground truth pixels and predicted pixels respectively.

In the classification task, we computed accuracy [35], precision [35], recall [35], F1‐score [35] to evaluate our performance. They can be calculated as follows:

(6) $$\text{Accuracy}=\frac{\text{TP}+\text{TN}}{\text{TP}+\text{FP}+\text{TN}+\text{FN}}$$

(7) $$\text{Recall}=\frac{\text{TP}}{\text{TP}+\text{FN}}$$

(8) $$\text{Precision}=\frac{\text{TP}}{\text{TP}+\text{FP}}$$

(9) $$F1-\text{Score}=\frac{2\times \text{Precision}\times \text{Recall}}{\text{Precision}+\text{Recall}}$$

where TP,TN,FP, and FN are the numbers of true positive, true negative, false positive, and false negative, respectively.

### 3.3 Segmentation results

Table  4 shows the segmentation result between our proposed model and the U‐Net and U‐Net++ model. We segmented all three types of lesions respectively to facilitate the comparison between each lesion.

**TABLE 4:** Results of segmentation between different models in different type lesions

Model	Lesion	Dice	Pixel precision	Pixel recall
U‐Net	Fibrosis	0.833	0.897	0.851
	Consolidation	0.735	0.844	0.787
	GGO	0.780	0.805	0.822
U‐Net++	Fibrosis	0.879	0.919	0.899
	Consolidation	0.820	0.873	0.862
	GGO	0.829	0.850	0.897
Ours	Fibrosis	**0.901**	**0.934**	**0.940**
	Consolidation	**0.864**	**0.872**	**0.913**
	GGO	**0.860**	**892**	**0.910**

From Table  4, we can see that compared with U‐Net and U‐Net++, our model gets the best performance in all lesions in all evaluation metrics. In this task, the performance of U‐Net is not excellent. U‐Net++ results also get a significant improvement than U‐Net. Fibrosis is the easiest to segment compared with other lesions. Consolidation and ground‐glass opacity are more complex than fibrosis. Our model is designed specifically for these lesions that is hard to segment and get greatly improved in dice, pixel recall and pixel precision.

### 3.4 Severity of COVID‐19 analysis

**FIGURE 4:**
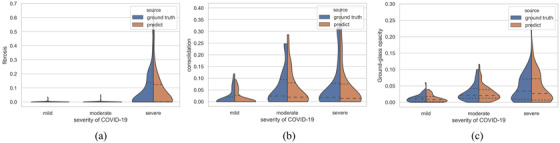
The relation diagram between different types of lesions and the severity of the disease, the abscissa is the severity of the disease, and the ordinate is the ratio area of the lesion in the lung area. the (a), (b), and (c), in turn, represent fibrosis, consolidation, and ground‐glass opacity. the ground truth is represented by blue on the left and the predict result is represented by brown on the right

In addition to locating various types of lesions accurately, we can eventually provide the proportion of different types of lesions in the lung area to get a preliminary diagnosis of the severity. The statistics can be seen in Figure  4 and Table  5.

**TABLE 5:** The *p*‐value of the predicted proportion of lesion area and severity of COVID‐19

Lesion type	Fibrosis	Consolidation	GGO
p‐value	1.2e‐5	9.0e‐3	3.1e‐4

As can be seen from Figure  4 and Table  5, the result we predicted is generally the same as the ground truth. Different severity of COVID‐19 with different sizes of the lesion area ratio distributes range in the same type of lesions. The area ratio of different kinds of lesions often predicts the severity of the disease. The p‐value of the predicted proportion of lesion area and severity of COVID‐19 is really small (*p*‐value <0.01), which means there is a strong correlation between the predicted proportion of lesion area and severity of COVID‐19. As we can see from Figure  4, fibrosis is rarely present in mild or moderate patients, so it is usually a sign that the patient is seriously infected when it occurs in the CT slices. For other types of lesions, the larger the lesion's area ratio means the more severe the disease is. This indicates that it is necessary for us to predict the segmentation of various kinds of lesions. We can have a preliminary judgment of the severity of COVID‐19 and give a more appropriate treatment plan according to the initial judgment through the segmentation results.

### 3.5 Classification result

As shown in Table  6, we achieve an accuracy of 98.3% at the human level in diagnosing whether a person is infected with COVID‐19. Besides the accuracy, recall is also crucial in diagnosing patients with COVID‐19 because a low recall can cause patients with COVID‐19 to flow into the community and lead to re‐infection. The classification model achieves a recall of 98.7%, which means only a few patients with COVID‐19 will be missed.

**TABLE 6:** Performance comparisons on SE‐ResNet34 and our classification model in distinguishing patients with COVID‐19

Model	Accuracy	Recall	Precision	F1‐score
Encoder of segmentation	**0.983**	**0.987**	**0.974**	**0.980**
SE‐ResNet34	0.875	0.889	0.806	0.846

**FIGURE 5:**
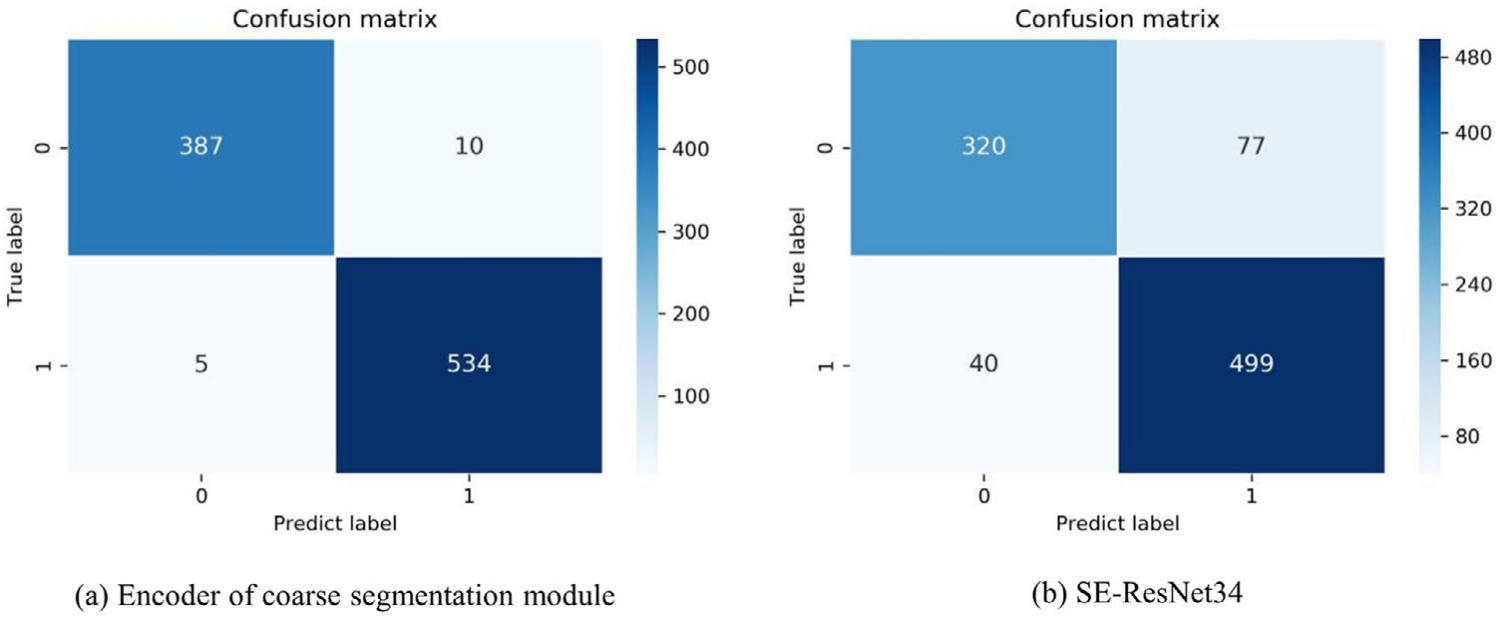
The confusion matrix on the five‐fold test set for diagnosing whether a person is infected with COVID‐19. the person that predicted without COVID‐19 is represented by 0, and the patient with COVID‐19 is represented by 1. (a) represent the classification model is the encoder of the coarse segmentation model, and (b) mean the classification is the SE‐ResNet34

By comparing the results of our classifier with the SE‐ResNet34 model, we can find that encoder of coarse segmentation module trained with segmentation annotation data has a stronger ability to extract features, which can significantly improve the classification performance of the encoder. As a kind of supervision information, the segmentation annotation is also passed to the encoder through the loss function of segmentation. The classifier trained with segmentation annotation data enhances the accuracy and recalls by nearly ten percentage points, while precision also improves by nearly seventeen percentage points. The confusion matrix is shown in Figure  5.

We adopt the Friedman test [36] for the classification results due to the quantity of the two classes are really small. Specifically, we believe that the person is infected with COVID‐19 when the predicted probability is greater than 0.5. The prediction result is either 1 or 0 for everyone. The patient with COVID‐19 is represented by 1, and the patient without COVID‐19 is represented by 0 in the ground truth. We conduct Friedman tests on results of SE‐ResNet34 against the real label, and the result is 1.463. We also conduct Friedman tests on the results of our method against the real label, and the result of the test is 0.308. The critical value [36] at p<0.05 for the data with a sample size of 936 and the number of attributes of 2 is 59.97. Obviously, the Friedman test results on SE‐ResNet34 and our method are much smaller than the critical value, which illustrates only little difference between the predicted results and the truth label.

### 3.6 Ablation study

In addition to the mentioned results above, we are also interested in how each component in the model affects the result. Accordingly, we quantified how each module improved the results. Therefore, we added a submodule to the original model and observed the effect of each module. The ablation studies were conducted in the same experimental environment to make sure a fair experimental comparison. Here, we grouped all lesions into one category for calculation and judged the quality of results by comparing dice to facilitate the comparison of results. From Table  7, it is evident that all components in our model can improve the results. It can be seen from Table  7 that adding a residual refine module can improve the result the most, nearly improved by 3.3%. The ASPP part can improve the result about 2.4%, and the hybrid loss can improve the result about 2.0%.

**TABLE 7:** The improvement of each component in our model

Model	Segmentation dice	Classification accuracy
U‐Net (SE‐ResNet34)	0.793	0.933
U‐Net (SE‐ResNet34) + ASPP (coarse segmentation module)	0.817	0.950
coarse segmentation module + refine module	0.850	0.961
coarse segmentation module + refine module + hybrid loss	**0.870**	**0.983**

The result improved by residual refine module proved that the coarse‐refine network is available. The result enhanced by the ASPP also indicates that ASPP can enhance the global information extraction ability and strengthen the ability to detect objects of different scales in the COVID‐19 segmentation task. The result improved by hybrid loss shows hybrid loss can accurately predict the delicate structures and clear boundaries. At the same time, each module has excellent help for the improvement of classification accuracy.

### 3.7 Case study

**FIGURE 6:**
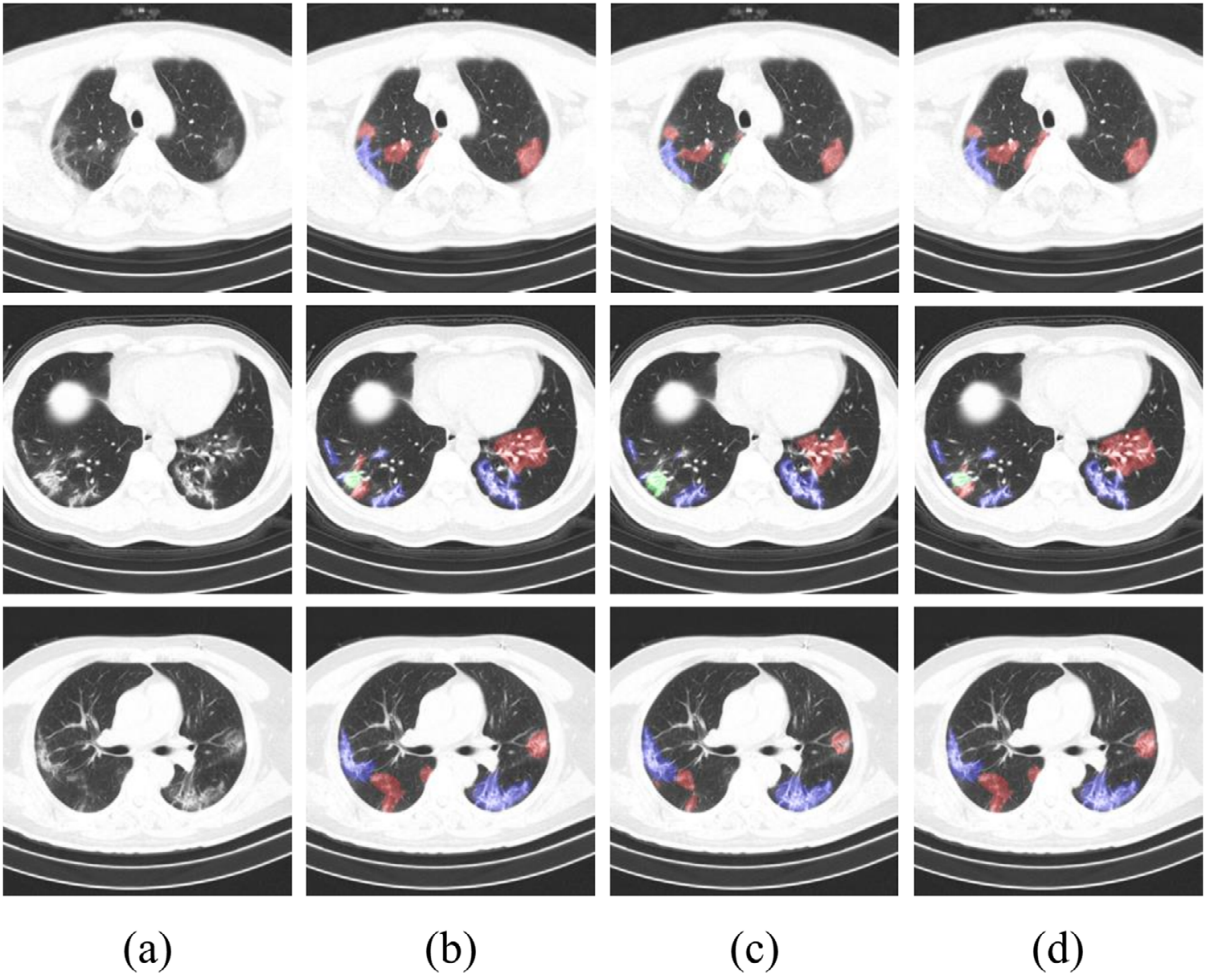
The visualisation of the segmentation results. blue, green, and red represent fibrosis, consolidation, and ground‐glass opacity, respectively. column (a) are the original images, column (b) are the contour labels of the lesion regions given by the doctors, column (c) is the segmentation result of U‐Net which dice is 0.793 and the accuracy is 0.856, and column (d) is our result which dice is 0.870, and the accuracy is 0.983

As an example, Figure  6 shows the segmentation results between U‐Net and our method. The results of our model compared with U‐Net, U‐Net ++ indicate the effectiveness of our method in the segmentation of COVID‐19. The pixel recall results of our approach are the best on all types of lesions, meaning that our model has a significant effect on areas that are difficult to be segmented, such as boundaries. The performance of U‐Net on the ground‐glass opacity and consolidation is not well, maybe because the consolidation scale is small and the ground‐glass opacity of contrast ratio is lower. U‐Net is not good at solving these kinds of problems. On the contrary, the U‐Net performs pretty well in fibrosis which has a clear boundary and big scales. Our model can solve the above problems better and get greater results than other medical image segmentation models.

It can be found from the comparison of segmentation results in Figure  6 that the results of our model are indeed much better than U‐Net in localisation and detection effect of infected areas with low contrast and small size.

## 4 DISCUSSION AND CONCLUSION

In this paper, we propose a coarse‐refine network for the CT lesion segmentation of the COVID‐19. Experimental results show that our model in the segmentation of COVID‐19 CT images outperforms other familiar medical segmentation models, enabling the doctor to get a more accurate estimate on the progression of the infection and thus can provide more reasonable treatment options. In the previous work, various U‐Net and their variants have been developed, achieving reasonable segmentation results in COVID‐19 applications. Military et al. [37] propose the V‐Net, which utilises the residual blocks as the basic convolutional block and optimises the network by a Dice loss. By equipping the convolutional blocks with the so‐called bottleneck blocks, Shan et al. [16] use a VB‐Net for more efficient segmentation. Oktay et al. [38] propose an attention U‐Net that is capable of capturing fine structures in medical images, thereby suitable for segmenting lesions and lung nodules in COVID‐19 applications. Inf‐Net [20] can automatically recognise infected regions from CT. It improves the quality of boundary segmentation. However, the above methods often regard all COVID‐19 lesions as one category, which cannot give the severity of a patient's infection according to the occurrence of many different lesions. Therefore, these methods can't provide more reasonable treatment options. Besides, the above methods do not well consider the differences between COVID‐19 lesions and other segmentation tasks, making COVID‐19 lesions difficult to segment. The main differences between COVID‐19 lesions and other segmentation tasks are as follows: some boundaries of infected regions are not evident [20, 22]; some areas of infection have low contrast with natural areas [21]; the shape and size of infected areas are multi‐scale [21, 23]. We propose a model to address those difficulties. We use a coarse segmentation module to obtain a rough segment result from input images. We employ a refining module to learn the residuals between the rough segment result and the ground truth to adjust the erroneous segmentation output, especially the edge. Moreover, we add an atrous spatial pyramid pooling module to both the coarse segmentation module and the refine module so that the network can accurately detect infected regions with different scales. We adopt the hybrid loss [25] as training loss, which helps guide the model to predict the delicate structures with clear boundaries accurately. However, our method also has its limitations. The structure of our network is relatively heavy, and the parameters of the model are relatively large, which requires more corresponding computing resources than some other methods. We believe that this method is universal and can achieve good results in other segmentation tasks. We will try this method in other fields in the future.
